# Simultaneous ventilation of two simulated ARDS patients in COVID-19 pandemic

**DOI:** 10.1186/s13054-020-02940-4

**Published:** 2020-05-11

**Authors:** Danny Epstein, Yoav Hoffman, George Dahoud, Aeyal Raz, Asaf Miller

**Affiliations:** 1 Internal Medicine “B” department, Rambam Health Care Campus, HaAliya HaShniya St. 8, 3109601 Haifa, Israel; 2 Pediatric Intensive Care unit, Galilee Medical Center, Nahariya, Israel; 3 Critical Care division, Rambam Health Care Campus, Haifa, Israel; 4 Department of Anesthesiology, Rambam Health Care Campus, Haifa, Israel; 5 Ruth and Bruce Rappaport Faculty of Medicine, Technion, Haifa, Israel; 6 Medical Intensive Care unit, Rambam Health Care Campus, Haifa, Israel

## Introduction

The COVID-19 pandemic created a shortage of ventilators in many parts of the world. Models predict that the number of patients that will require a ventilator ranges between 1.4 and 31 patients per available ventilator [1]. Given this potential, numerous groups have proposed modification of ventilator circuit to enable using a single ventilator to support multiple patients. Previous works demonstrated the feasibility of this method in models of healthy lungs, animals, and healthy volunteers [2–4]. In the current study, we used lung models with varying compliances, to investigate whether such simultaneous ventilation is feasible.

## Methods

**Fig. 1:**
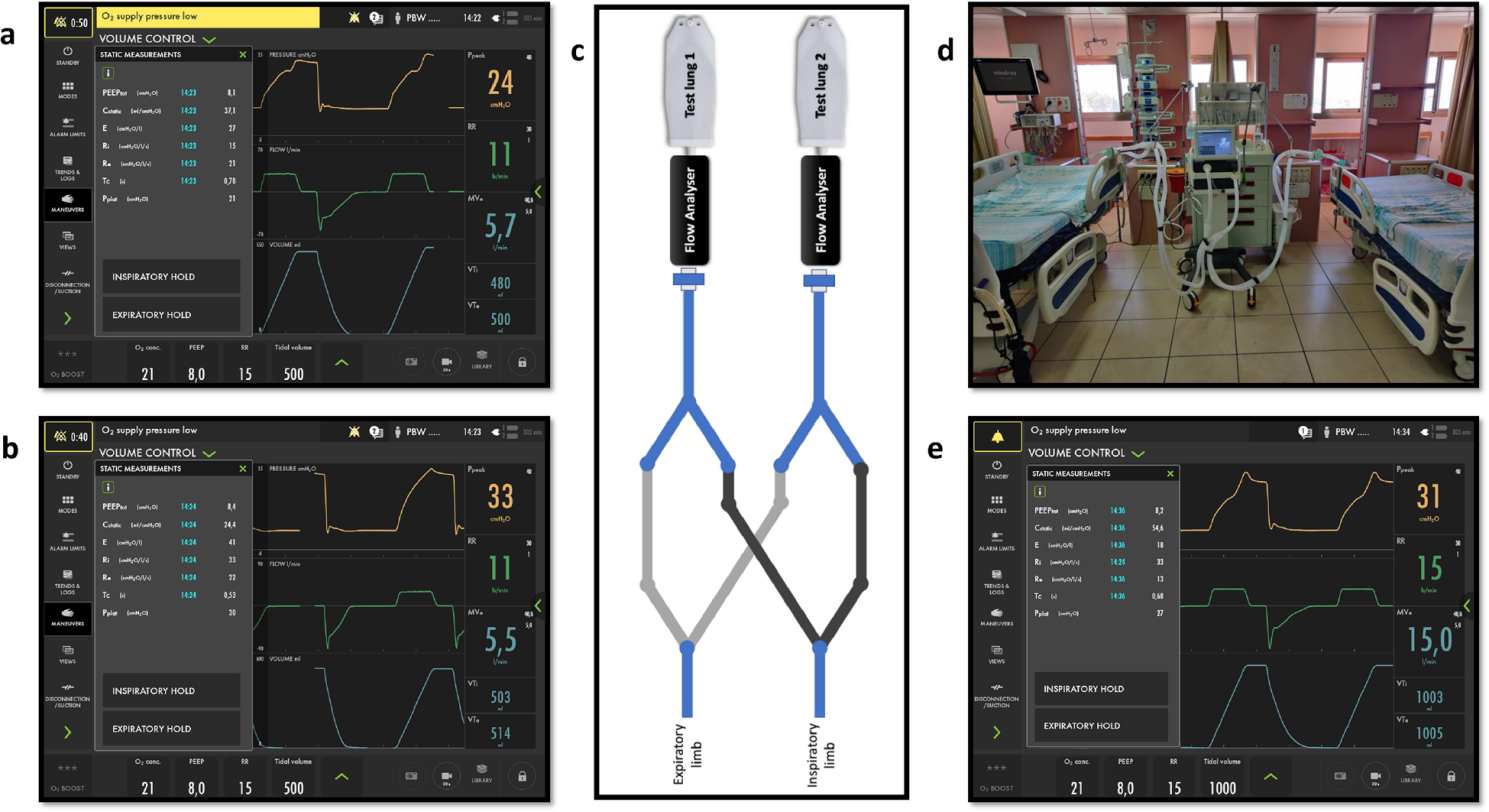
Simultaneous ventilation model. **a** Ventilation parameters of high-compliance lung (one lung ventilated). **b** Ventilation parameters of low-compliance lung (one lung ventilated). **c** Two-subject ventilator circuit (scheme). **d** Two-subject ventilator circuit (photography). **e** Combined ventilation parameters (both lungs connected)

The inspiratory and expiratory limbs of a Servo Air (Maquet©, Solna, Sweden) ventilator were split using Y-connectors to create two parallel circuits (Fig. 1). These were connected to two test lungs. The same ventilator connected separately to each lung was used to evaluate the compliance of each test lung. The measured compliances were 37 ml/cmH_2_O and 24 ml/cmH_2_O (Fig. 1). These correspond to compliance previously described in COVID-19 patients [5]. We used volume control and pressure control modes set to 1000 mL tidal volume (T_V_; 500 mL per lung) and 20 cmH_2_O above positive end-expiratory pressure (PEEP), respectively. We used the respiratory rate of 15 breaths/min and PEEP of 8 cmH_2_O, and peak pressure alarm was set to 40 cmH_2_O. We monitored the T_V_ and peak pressure of the combined lungs with the ventilator and of each lung separately using a FlowAnalyser PF-300 (Imtmedical©, Buchs, Switzerland). To evaluate the effects of complications such as tube blockage, we recorded the alarms during one lung obstruction.

## Results

Connected to the described configuration, the ventilator did not alarm, and both test lungs expanded. The pressures and volumes measured are shown in Table 1. The combined system compliance was 54.6 ml/cmH_2_O (Fig. 1); not surprisingly, the T_V_s were unevenly distributed between the test lungs.

**Table 1:** The combined and the individual pressure and volume characteristics of lung simulators

	Combined	Test lung 1 (compliance of 37 ml/cmH_2_O)	Test lung 2 (compliance of 24 ml/cmH_2_O)
Volume control (T_V_ = 1000 ml)			
**Tidal volume** (ml)	1000	473	314
**Positive end-expiratory pressure** (cmH_2_O)	8	8	8
**Peak pressure** (cmH_2_O)	30	31	31
Pressure control (PC = 20 cmH_2_O above PEEP)			
**Tidal volume** (ml)	1012	475	333
**Positive end-expiratory pressure** (cmH_2_O)	8	8	8
**Peak pressure** (cmH_2_O)	28	21	30

During a blockade trial of a single test lung, while ventilated on volume control mode, a “high pressure” alarm was recorded, whereas while performing this trial under pressure control, no alarm was recorded. Ten percent of total T_V_ did not reach the lungs due to increased dead space.

## Discussion

The overwhelming number of COVID-19 patients with respiratory failure leads to tremendous efforts to increase ventilation capacity worldwide. Under such conditions, the standards of care for an individual patient may be reduced to allow caring for more patients. However, we found that simultaneous ventilation of patients with different lung compliance prevents appropriate monitoring of pulmonary mechanics, T_V_, plateau, and driving pressures. This may preclude safe lung-protective ventilation. As the lung compliance varies greatly in different patients with respiratory failure, simultaneous ventilation of two or more patients with significant differences of their lung physiology may lead to major differences in the delivered T_V_s. A possible solution would be to assign patients to common ventilators based on lung compliance. However, this seems very complicated and time-consuming. Furthermore, even if applied, patients may deteriorate or recover at different rates causing previously similar lungs to drift apart.

Alarm monitoring, a critical safety measure of ventilators, is also impaired, especially when pressure control is used. Although not tested in our experiment, it seems that simultaneous ventilation of multiple patients would necessitate the usage of muscle relaxants as sensing patient effort and trying to synchronize the ventilation to such effort would be pointless under such circumstances.

Based on our preliminary findings, we conclude that simultaneous ventilation of patients with acute respiratory distress syndrome should be abandoned in favor of alternative methods to increase ventilator support capacity. It may be used only temporarily and as a last resort. Our findings support the recommendation of the American College of Chest Physicians [6].

## Data Availability

All data generated or analyzed during this study are included in this published article.

## Abbreviations

T_V_: Tidal volume
PEEP: Positive end-expiratory pressure
